# Multiple Comorbidities, Psychiatric Disorders, Healthcare Resource Utilization and Costs Among Adults with Essential Tremor: A Retrospective Observational Study in a Large US Commercially Insured and Medicare Advantage Population

**DOI:** 10.36469/001c.37307

**Published:** 2022-08-15

**Authors:** Dingwei Dai, Ali Samiian, Joaquim Fernandes, Henriette Coetzer

**Affiliations:** 1 CVS Health Clinical Trial Services LLC, Woonsocket, Rhode Island; 2 Cala Health, Inc., San Mateo, California

**Keywords:** essential tremor, comorbidity, multimorbidity, psychiatric disorders, healthcare resource utilization, healthcare costs, observational study

## Abstract

**Background:** Essential tremor (ET), the most common movement disorder, often impairs patients’ ability to perform activities of daily living, mental health, and quality of life.

**Objectives:** To assess comorbidities, psychiatric disorders, healthcare resource utilization (HCRU), and costs among patients with ET compared with patients without ET.

**Methods:** This retrospective observational study was conducted using a large US administrative claims database. Patients with ET were identified during the study period (1/1/2017–12/31/2019). The earliest claim date with ET diagnosis was identified as the index date. An index date was assigned randomly for each non-ET patient. Patients had to be at least 22 years old and be enrolled in the health plan for at least 6 months before and at least 12 months after the index date. Patients with and those without ET were matched 1:1 on age, gender, payer type, and first 3 digits of their ZIP code. Comorbidities were assessed using data within 6 months prior to the index date. Psychiatric disorders, HCRU, and costs were examined using data within 12 months after the index date.

**Results:** The mean (SD) age of ET patients (n = 5286) was 70.8 (11.8) years, 49.1% were female, and 82.9% were Medicare Advantage members. In the 12 months following the index date, 26.0% of patients had no insurance claims for ET-related pharmacotherapy or invasive therapies. Patients with ET had a higher number of comorbidities than non-ET patients (5.3 [3.2] vs 4.0 [3.3]); a higher prevalence of psychiatric disorders (depression: 25.6% vs 15.3%; adjusted odds ratio (AOR) [95% CI], 1.56 [1.41-1.73]; anxiety: 27.7% vs 15.5%, AOR: 1.78 [1.61-1.96]); and higher total healthcare costs: $17 560 [$39 972] vs $13 237 [$27 098], adjusted cost ratio [95% CI]: 1.11 [1.06-1.16]; all *P*<.0001.

**Discussion:** Highly prevalent multiple comorbidities and psychiatric disorders should be considered in the context of clinical decision-making to optimize ET management.

**Conclusions:** This study represents the largest observational study to report ET disease and economic burdens in a real-world setting. The data demonstrate increased comorbidity, mental health, and healthcare cost burdens among ET patients compared with matched non-ET patients. These findings underscore the need for innovative care for this complex population.

## INTRODUCTION

Essential tremor (ET) is one of the most common neurological movement disorders in adults.^1,2^ Essential tremor prevalence is around 4% in persons age 40 and older and increases considerably with age, affecting an estimated 20% of individuals in their 90s and over worldwide.^3^ A new meta-analysis demonstrated that the pooled prevalence for all ages was 1.33% worldwide, and gender did not impact the prevalence of ET.^4^ Louis et al^5^ reported that ET affects approximately 2.2% of the US population, or 7 million individuals in the United States. Recently published analysis of claims data from 819 661 patients in the United States found that the ET prevalence rate increased at an annual growth rate of 6.27% from 2010 to 2018, with 7.29% and 2.05% annual growth rates for the Medicare Advantage and commercially insured populations, respectively.^6^

While traditionally regarded as benign, ET is in fact a chronic, progressive, and disabling neurological disease that causes uncontrolled rhythmic shaking, most often affecting the hands.^7–9^ It is characterized by the presence of an action tremor as well as a myriad of symptoms such as gait disturbances, postural instability, and cognitive impairment.^7,9^ These symptoms may worsen over time and significantly impact a patient’s quality of life and ability to perform activities of daily living.^2,10–12^

Medications used to treat ET can be moderately effective at reducing symptoms but do not treat the underlying disease.^13,14^ It is estimated that only 30% to 50% of patients with ET respond to pharmacotherapy.^11,15^ Invasive therapies include deep brain stimulation (DBS), magnetic resonance–guided focused ultrasound (MRgFUS), and thalamotomy for unilateral and DBS for bilateral procedures.^13,16^ Only 3% of patients with ET whose tremors are refractory to pharmacotherapy choose to undergo DBS.^17^ Both thalamotomy and DBS have been shown to be highly effective at reducing limb tremor magnitude; however, they are invasive procedures and associated with significant risks of side effects.^18,19^ A newly developed surgical alternative, MRgFUS, may be ideal for patients with substantial comorbidities but can be applied to only a very limited number of patients with ET due to the selective criteria. In addition, because it involves ablation of brain tissue, MRgFUS may result in permanent side effects.^13,20^ Recent research has explored novel noninvasive, nonpharmacological treatment, such as noninvasive transcutaneous patterned afferent stimulation to aid in the symptomatic relief of hand tremor; these therapies may mitigate some of the existing treatment gaps.^2,16,21–23^

There is limited real-world evidence on the burden of comorbidities, mental disorders, healthcare resource utilization (HCRU), and healthcare costs (HCC) among patients with ET. Such evidence is important for clinical and policy decision-making to optimize ET care and improve outcomes. This study aimed to assess the comorbidity burden, prevalence of psychiatric disorders, HCRU, and HCCs among adult patients with ET in the commercially insured and Medicare Advantage populations using a large US administrative claims database.

## METHODS

### Study Design and Data Source

A retrospective observational cohort study of adult patients with ET was conducted using a large US administrative claims data from January 1, 2017, to December 31, 2019 (study period). The claims database included over 20 million medical insurance beneficiaries and contained patient enrollment data, as well as inpatient and outpatient medical and pharmacy claims for fully insured commercial health plan and Medicare Advantage members.^24^ All data handling complied with federal and state requirements; the privacy and security of individually identifiable personal health information, required by the Health Insurance Portability and Accountability Act (HIPAA), were preserved. The study was approved by an independent institutional review board prior to initiation.

### Patient Selection

Patients with ET were identified between July 1, 2017, and December 31, 2018 (index period). Patients were considered to have ET if they had at least 1 medical claim with an ET diagnosis code (*International Classification of Diseases, Tenth Revision, Clinical Modification* [ICD-10-CM] code of G25.0 in any position). The first observed ET diagnosis date was defined as the index date. An index date was assigned randomly for each patient without ET in the database. All patients were at least 22 years of age at the index date and had fully insured commercial health and/or Medicare Advantage coverage with medical and pharmacy health insurance benefits for at least 6 months prior to the index date (baseline period) and 12 months after the index date (follow-up period). Patients with a diagnosis of Parkinson’s disease (ICD-10-CM: G20.x) or thyroid disorders (ICD-10-CM: E00.x–E03.x, E06.5, E07.9, E89.0, or thyroid hormone prescription) were excluded from the ET cohort. A non-ET comparison cohort was created using 1:1 exact matching on age, gender, payer type, first 3-digits of ZIP code, and index month.

### Demographic Characteristics

Patient demographic characteristics included age at index date, gender, geographic region (Midwest, Northeast, South, and West), rural or urban residence, and median household income. Household income was estimated by merging 2010 census data to the claims data using ZIP code.

### Assessment of Comorbidities

The 47 most common chronic conditions in the Aetna administrative claims data were identified using previously described methods based on ICD-10-CM codes (**Online Supplemental Material, Table S1**).^24,25^ We also calculated Charlson Comorbidity Index (CCI) and age-adjusted CCI, 2 validated metrics that summarize disease burden and predict mortality and high HCC risk.^26–29^ All comorbid conditions were assessed using all claims data within 6 months prior to the index date.

### Assessment of Psychiatric Disorders

Psychiatric disorders were identified using ICD-10-CM codes (**Online Supplemental Material, Table S2**) during the 12-month follow-up period.

### ET Treatment

Patients with ET were categorized into 1 of 3 treatment groups consistent with evidence of billed treatments in the claims database during the 12-month follow-up period: (1) *pharmacotherapy*, those patients given evidence of filled prescription for propranolol and other β-blockers, primidone, anti-epileptic drugs, benzodiazepines, or botulinum neurotoxin; (2) *invasive therapy*, those given evidence of DBS, thalamotomy, focused ultrasound, radio-surgical gamma knife thalamotomy; or (3) *untreated*, those who did not receive any ET-related pharmacotherapy or invasive therapy.

### Healthcare Resource Utilization and Costs

Healthcare resource utilization included any emergency department (ED) visits, inpatient admission, and length of hospital stays (LOS) among those with inpatient admissions. All-cause HCRU was assessed using all medical claims; ET-related HCRU was assessed using all medical claims with a primary diagnosis of ET (ICD-10-CM: G25.0). All-cause HCC included costs of all medical claims and pharmacy claims; ET-related HCC included costs of medical claims with a primary diagnosis of ET and ET-related pharmacotherapy costs of pharmacy claims. HCRU and HCC were aggregated over the 12-month follow-up period. All HCC were inflated to 2019 US dollars using the medical care services component of the Consumer Price Index.^30^

### Statistical Analysis

Demographic and clinical characteristics and comorbidities were analyzed descriptively. Means (±SD) or medians (interquartile range) were reported for continuous variables, and frequencies (%) were reported for categorical variables. To compare differences between treatment groups, age groups (<65, ≥65 years old), and ET and non-ET groups, statistical significance was assessed with the Student *t* test, Wilcoxon rank-sum test, or Kruskal-Wallis test for continuous variables, and the Pearson χ^2^ test for categorical variables. To evaluate the association of patient characteristics and the HCC, generalized linear models were performed to calculate adjusted cost ratios with the corresponding 95% confidence interval (CI). Log-transformation and gamma distribution were applied based on the distribution and presence of heteroskedasticity.^24,31^ To evaluate the association of ET and the prevalence of psychiatric disorders and HCC, generalized linear models were performed to calculate adjusted odds ratios and adjusted cost ratio with the corresponding 95% CI, respectively. All data management and statistical analyses were conducted using SAS version 9.4 statistical software (SAS Institute Inc, Cary, North Carolina). All *P* values were 2-sided, with *P* < .05 considered statistically significant.

## RESULTS

### Patient Characteristics

Of the 5286 eligible patients with ET (**Online Supplemental Material, Figure S1**), 49.1% were female; median age was 72 years, with 79% over the age of 65; 37.5% were in the southern United States; and 50.4% lived in rural areas (Table 1). Medicare Advantage patients made up 82.9% of the study population; the remaining 17.1% were commercially insured patients. Among patients with ET, 71.3% received some pharmacotherapy (pharmacotherapy group), 26.0% received no ET-related treatment (untreated group), and 2.7% had invasive therapies (invasive therapy group) during the 12-month follow-up period. Patients with invasive therapy were younger (untreated, 72.0 (SD = 11.3); pharmacotherapy,70.5 (SD=11.9); invasive therapy, 67.2 (SD = 12.3); *P* < .0001), more female (untreated, 45.6%; pharmacotherapy, 49.7%; invasive therapy, 69.7%; *P* < .0001), and had a smaller age-adjusted CCI: untreated 4.27 (SD=2.62); pharmacotherapy, 4.41 (SD=2.77); invasive therapy, 3.75 (SD=2.30), *P*=.0161).

**Table 1.** Patient Demographic and Clinical Characteristics of Patients with ET at Baseline by Treatment Group<sup>a</sup>

		**Patients with ET**				**Patients without ET (n-5244)**	
**Characteristics**	**Overall (n=5286)**		**Treatment Group**				***P* Value^c^**
		**Untreated (n=1374)**	**Pharmacotherapy (n=3767)**	**Invasive Therapy (n=145)**	***P* Value^b^**		
Age (y)							
Mean (SD)	71 (11.81)	72.03 (11.28)	70.46 (11.93)	67.25 (12.32)	<.0001	71 (11.78)	.8603
Age group (y)							
22-44	197 (3.73)	44 (3.2)	141 (3.74)	12 (8.28)	<.0001	184 (3.51)	.8014
45-64	890 (16.84)	165 (12.01)	693 (18.4)	32 (22.07)		896 (17.09)	
≥65	4199 (79.44)	1165 (84.79)	2933 (77.86)	101 (69.66)		4164 (79.41)	
Gender, n (%)							
Male	2690 (50.89)	747 (54.37)	1895 (50.31)	48 (33.1)	<.0001	2668 (50.88)	.9902
Female	2596 (49.11)	627 (45.63)	1872 (49.69)	97 (66.9)		2576 (49.12)	
Geographic region							
Midwest	1565 (29.61)	376 (27.37)	1147 (30.45)	42 (28.97)	<.0001	1550 (29.56)	.9945
Northeast	1398 (26.45)	477 (34.72)	887 (23.55)	34 (23.45)		1391 (26.53)	
South	1984 (37.53)	439 (31.95)	1487 (39.47)	58 (40.)		1973 (37.62)	
West	339 (6.41)	82 (5.97)	246 (6.53)	11 (7.59)		330 (6.29)	
Urban-rural							
Urban	1097 (20.75)	300 (21.83)	768 (20.39)	29 (20.0)	.2912	1101 (21.0)	.8773
Suburban	1523 (28.81)	414 (30.13)	1064 (28.25)	45 (31.03)		1489 (28.39)	
Rural	2666 (50.44)	660 (48.03)	1935 (51.37)	71 (48.97)		2654 (50.61)	
Median household income ($)							
Mean (SD)	61 394 (22 040)	62 916 (22 781)	60 838 (21 745)	61 95 (22 001)	.0332	60 368 (21 955)	.0254
Median (IQR)	56 724 (45 826- 71 988)	57 003 (47 143- 73 467)	56 612 (45 459- 71 244)	56 894 (46 232- 72 882)	.0332	56 084 (44 650- 71 570)	.0937
Quartile 1, <$45 725	1322 (25.01)	313 (22.78)	974 (25.86)	35 (24.14)	.1319	1440 (27.46)	.0424
Quartile 2, $45 726-$56 659	1321 (24.99)	360 (26.20)	923 (24.50)	38 (26.21)		1263 (24.08)	
Quartile 3, $56 660-$71 948	1324 (25.05)	329 (23.94)	961 (25.51)	34 (23.45)		1269 (24.2)	
Quartile 4, >$71 948	1319 (24.95)	372 (27.07)	909 (24.13)	38 (26.21)		1272 (24.26)	
Payers							
Commercial insurance	902 (17.06)	222 (16.16)	650 (17.26)	30 (20.69)	.2912	887 (16.91)	.8383
Medicare Advantage	4384 (82.94)	1152 (83.84)	3117 (82.74)	115 (79.31)		4357 (83.09)	
CCI, mean (SD)	1.7 (2.29)	1.49 (2.17)	1.79 (2.34)	1.44 (1.83)	<.0001	1.51 (2.22)	<.0001
ACCI, mean (SD)	4.36 (2.72)	4.27 (2.62)	4.41 (2.77)	3.75 (2.3)	.0161	4.17 (2.68)	<.0001
No. of comorbidities							
Mean (SD)	5.26 (3.21)	4.77 (2.998)	5.44 (3.26)	5.33 (3.47)	<.0001	4.03 (3.27)	<.0001
Median (IQR)	5.0 (3 - 7)	4.0 (3 - 7)	5.0 (3 - 7)	5.0 (3 - 7)	<.0001	4.0 (1-6)	<.0001
No. of comorbidities group							
≤4	2435 (46.07)	712 (51.82)	1658 (44.01)	65 (44.83)	<.0001	3170 (60.45)	<.0001
5-7	1647 (31.16)	422 (30.71)	1179 (31.3)	46 (31.72)		1302 (24.83)	
≥8	1204 (22.78)	240 (17.47)	930 (24.69)	34 (23.45)		772 (14.72)	

Abbreviations: ACCI, age-adjusted Charlson Comorbidity Index; CCI, Charlson Comorbidity Index; ET, essential tremor; IQR, interquartile range. ^a^Unless otherwise noted, all data are reported as number (%) of patients. ^b^*P* value for comparison among treatment groups. ^c^*P* value for overall comparison between patients with ET and those without ET.

### Comorbidity and Multimorbidity

**Figure 1.**
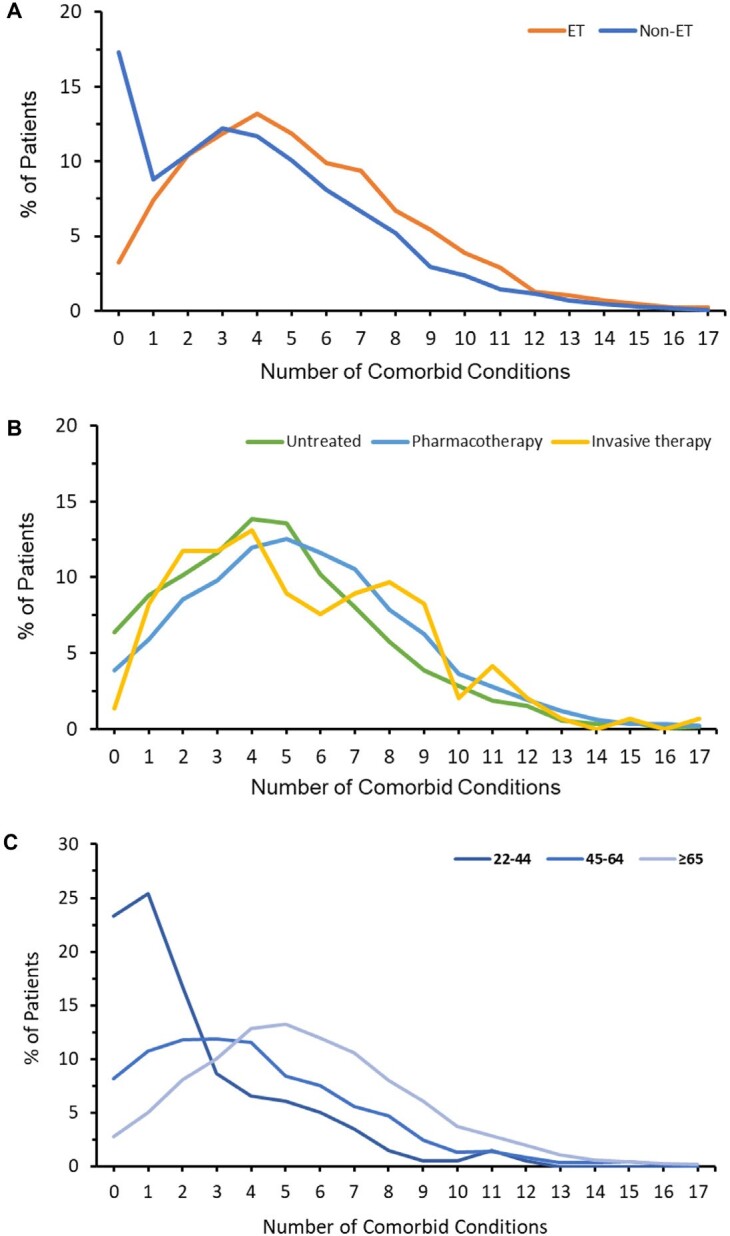
Distribution of Number of Comorbid Conditions Distribution of number of comorbid conditions in patients with and without ET (**A**) and in patients with ET by treatment type (**B**) and age group (**C**). Abbreviation: ET, essential tremor.

The 5 most common comorbidities were hypertension (67.9% of patients with ET), pain disorders (61.8%), hyperlipidemia (55.4%), fatigue and sleep-related disorders (28.1%), and diabetes mellitus (27.3%) (see Table 2 for the complete list of top 30 most common comorbidities in the study population). The distribution of number of comorbid conditions in patients with ET varied by treatment type and age (Figure 1). Many patients had multiple comorbidities: 79.7% of patients with ET had at least 3 comorbidities, 56.9% had at least 5 comorbidities, and 23.4% had at least 8 comorbidities. Common comorbidities and multiple comorbidities patterns varied by age and by ET treatment type (Table 2; **Figure 1; Online Supplemental Material, Table S3**). The mean (SD) number of comorbidities for patients age 65 years or older (5.6 [3.1]) was higher than for patients age 45 to 64 (4.6 [3.3]) and those 45 years or under (2.4 [2.6]), *P* < .0001. The mean number of comorbidities varied by treatment type: untreated, 4.8 (3.0); pharmacotherapy, 5.4 (3.3); invasive therapy 5.3 (3.5); *P* < .0001.

**Table 2.** Prevalence of the 30 Most Common Comorbidities Among Patients With ET at Baseline by Treatment Group

		**Patients with ET**				**Patients without ET (n-5244)**	
**Comorbidity**	**Overall (n=5286)**		**Treatment Group**				***P* Value^c^**
		**Untreated (n=1374)**	**Pharmacotherapy (n=3767)**	**Invasive Therapy (n=145)**	***P* Value^b^**		
Hypertension	3587 (67.86)	894 (65.07)	2611 (69.31)	82 (56.55)	.0002	2981 (56.85)	<.0001
Pain disorders	3269 (61.84)	738 (53.71)	2425 (64.37)	106 (73.1)	<.0001	2386 (45.5)	<.0001
Hyperlipidemia	2928 (55.39)	760 (55.31)	2089 (55.46)	79 (54.48)	.9714	2266 (43.21)	<.0001
Fatigue and sleep related disorders	1485 (28.09)	351 (25.55)	1089 (28.91)	45 (31.03)	.0434	888 (16.93)	<.0001
Diabetes mellitus	1444 (27.32)	324 (23.58)	1085 (28.8)	35 (24.14)	.0007	1,291 (24.62)	.0016
Obesity	1366 (25.84)	330 (24.02)	1004 (26.65)	32 (22.07)	.0928	929 (17.72)	<.0001
Osteoarthritis	1334 (25.24)	304 (22.13)	983 (26.1)	47 (32.41)	.0019	972 (18.54)	<.0001
Anxiety	1174 (22.21)	201 (14.63)	931 (24.71)	42 (28.97)	<.0001	542 (1.34)	<.0001
Chronic pulmonary disease	1076 (20.36)	240 (17.47)	809 (21.48)	27 (18.62)	.0059	776 (14.8)	<.0001
Depression	1065 (20.15)	198 (14.41)	834 (22.14)	33 (22.76)	<.0001	540 (1.3)	<.0001
Ischemic heart disease	1020 (19.3)	262 (19.07)	738 (19.59)	20 (13.79)	.2148	914 (17.43)	.0134
Cancer (malignant)	674 (12.75)	177 (12.88)	484 (12.85)	13 (8.97)	.3827	585 (11.16)	.0117
Cerebrovascular disease	649 (12.28)	177 (12.88)	456 (12.11)	16 (11.03)	.6776	413 (7.88)	<.0001
Chronic kidney disease	630 (11.92)	155 (11.28)	457 (12.13)	18 (12.41)	.6946	561 (1.7)	.0481
Peripheral vascular disease	596 (11.28)	148 (10.77)	435 (11.55)	13 (8.97)	.4963	471 (8.98)	<.0001
Atrial fibrillation	591 (11.18)	148 (10.77)	427 (11.34)	16 (11.03)	.8498	505 (9.63)	.0092
Substance use disorders	570 (10.78)	104 (7.57)	451 (11.97)	15 (10.34)	<.0001	366 (6.98)	<.0001
Glaucoma	474 (8.97)	108 (7.86)	358 (9.5)	8 (5.52)	.0638	476 (9.08)	.8439
Congestive heart failure	471 (8.91)	112 (8.15)	347 (9.21)	12 (8.28)	.4799	439 (8.37)	.3251
Osteoporosis	461 (8.72)	123 (8.95)	322 (8.55)	16 (11.03)	.5464	298 (5.68)	<.0001
Diverticular disease	365 (6.91)	92 (6.7)	262 (6.96)	11 (7.59)	.899	257 (4.9)	<.0001
Rheumatoid arthritis	268 (5.07)	50 (3.64)	206 (5.47)	12 (8.28)	.0061	182 (3.47)	<.0001
Chronic thyroid disorders	264 (4.99)	75 (5.46)	178 (4.73)	11 (7.59)	.1967	796 (15.18)	<.0001
Dementia	249 (4.71)	78 (5.68)	170 (4.51)	1 (0.69)	.0149	185 (3.53)	.0023
Iron deficiency anemia	216 (4.09)	49 (3.57)	160 (4.25)	7 (4.83)	.4963	172 (3.28)	.0281
Epilepsy	184 (3.48)	59 (4.29)	116 (3.08)	9 (6.21)	.0211	59 (1.13)	<.0001
Inflammatory bowel disease	167 (3.16)	32 (2.33)	131 (3.48)	4 (2.76)	.1097	95 (1.81)	<.0001
Liver disease	165 (3.12)	34 (2.47)	129 (3.42)	2 (1.38)	.1054	134 (2.56)	.0803
Bipolar disorder	163 (3.08)	23 (1.67)	134 (3.56)	6 (4.14)	.0019	51 (.97)	<.0001
Kidney stones	159 (3.01)	43 (3.13)	109 (2.89)	7 (4.83)	.3898	94 (1.79)	<.0001

Abbreviation: ET, essential tremor. ^a^Unless otherwise noted, all data are reported as number (%) of patients. ^b^*P* value for comparison among treatment groups. ^c^*P* value for comparison between overall patients with ET and those without ET.

### Psychiatric Disorders

Prevalence of psychiatric disorders among patients with ET varied by treatment type and age group (**Table 3; Online Supplemental Material, Table S4**). Across the cohort, 2425 (45.9%) patients with ET had psychiatric disorders. The most common psychiatric disorders were anxiety (27.7% of patients with ET), depression (25.6%), and substance abuse disorders (13.8%). Many patients with ET (14%) had at least 2 psychiatric disorders. Psychiatric disorders were more common in the invasive therapy group than in the pharmacotherapy or untreated groups (prevalence of any psychiatric disorders: 57.9%, 49.2%, and 35.5%, respectively; *P* < .0001; Table 3). In contrast to the pattern seen with overall comorbidities, the prevalence of psychiatric disorders among patients of age 65 years or older (43.1%) was lower than age 45 to 64 (57.1%) and age 45 or under (54.8%), *P* < .0001 (**Supplemental Table S4**).

**Table 3.** Prevalence of Psychiatric Disorders Among Patients With and Without ET During the 12-Month Follow-up Period<sup>a</sup>

**Comorbidity**			**Patients With ET**			**Patients Without ET (n = 5244)**	
	**Overall (n = 5286)**		**Treatment Group**				***P* Value^c^**
		**Untreated (n = 1374)**	**Pharmacotherapy (n = 3767)**	**Invasive Therapy (n = 145)**	***P* Value^b^**		
Depression	1354 (25.61)	264 (19.21)	1035 (27.48)	55 (37.93)	<.0001	801 (15.27)	<.0001
Anxiety	1466 (27.73)	250 (18.2)	1159 (30.77)	57 (39.31)	<.0001	810 (15.45)	<.0001
Stress and adjustment disorders	278 (5.26)	54 (3.93)	214 (5.68)	10 (6.9)	.0303	168 (3.2)	<.0001
Dissociative and conversion disorders	31 (0.59)	6 (0.44)	24 (0.64)	1 (0.69)	.6973	10 (0.19)	.0011
Somatoform disorders	41 (0.78)	7 (0.51)	30 (0.8)	4 (2.76)	.0129	20 (0.38)	.0077
Substance use disorders	729 (13.79)	122 (8.88)	583 (15.48)	24 (16.55)	<.0001	551 (10.51)	<.0001
Any comorbid psychiatric conditions	2425 (45.88)	488 (35.52)	1853 (49.19)	84 (57.93)	<.0001	1630 (31.08)	<.0001

Abbreviation: ET, essential tremor. ^a^Unless otherwise noted, all data are reported as number (%) of patients). ^b^*P* value for comparison among treatment groups. ^c^*P* value for comparison between overall patients with ET and those without ET.

### Healthcare Resource Utilization

During the 1-year post-index period, 30.2% of patients with ET had at least 1 ED visit and 21.0% had at least 1 inpatient admission, with an average LOS per patient of 3.3 days. Invasive therapy patients had higher ED visit and inpatient admission rates and shorter LOS compared with pharmacotherapy and untreated patients (ED visit: 32.4%, 31.8%, and 25.6% respectively, *P* < .0001; inpatient admission: 24.8%, 22.4%, and 16.7%, respectively, *P* < .0001; LOS: 9.0 days, 14.3 days, and 10.6 days, respectively, *P* < .0001) (Table 4).

**Table 4.** All-Cause and ET-Related HCRU and HCCs Among Patients With and Without ET for the 12-Month Follow-up Period<sup>a</sup>

**Patients With ET**						**Patients Without ET (n=5244)**	
	**Overall (n = 5286)**		**Treatment Group**				***P*** **Value^b^**
		**Untreated (n = 1374)**	**Pharmacotherapy (n = 3767)**	**Invasive Therapy (n=145)**	***P*** **Value^b^**		
All-cause HCRU							
Any inpatient admission, n (%)	1111 (21.02)	230 (16.74)	845 (22.43)	36 (24.83)	<.0001	864 (16.5)	<.0001
Any ED visit, n (%)	1595 (30.17)	351 (25.55)	1197 (31.78)	47 (32.41)	<.0001	1313 (25.04)	<.0001
LOS, mean (SD)	3.31 (13.33)	2.46 (10.57)	3.64 (14.32)	2.78 (9.02)	<.0001	2.68 (11.81)	<.0001
All-cause HCCs ($), mean (SD)							
Inpatient admissions	5645 (30 705)	4598 (46 871)	5921 (22 120)	8386 (28 965)	<.0001	4168 (15 294)	<.0001
ED visit	916 (2824)	680 (1855)	986 (3085)	1361 (3184)	<.0001	635 (2233)	<.0001
PCP visits	621 (560)	556 (520)	640 (568)	735 (633)	<.0001	442 (448)	<.0001
Specialist visit	1348 (4506)	961 (1336)	1449 (5226)	2371 (3325)	<.0001	1049 (5237)	<.0001
Total medical	13 723 (36 836)	10 823 (50 401)	14 358 (30 96)	24 726 (36 888)	<.0001	10 308 (23 887)	<.0001
Total pharmacy^c^	3837 (11 550)	2432 (7625)	4289 (12 580)	5403 (13 256)	<.0001	2929 (9921)	<.0001
Total allowed costs^c^	17 560 (39 972)	13 255 (51 528)	18 647 (34 626)	30 130 (40 011)	<.0001	13 237 (27 098)	<.0001
ET-related HCRU							
Any inpatient admission, n (%)	374 (7.08)	84 (6.11)	276 (7.33)	14 (9.66)	0.1523	N/A	
Any ED visit, n (%)	198 (3.75)	40 (2.91)	151 (4.01)	7 (4.83)	0.1462	N/A	
LOS, mean (SD)	0.71 (5.14)	0.68 (4.18)	0.74 (5.54)	0.32 (01.42)	<.0001	N/A	
ET-related HCCs ($), mean (SD)							
Inpatient admissions	1296 (9947)	1040 (5406)	1346 (11 118)	2426 (10 843)	0.1455	N/A	
ED visit	122 (839)	87 (629)	133 (906)	148 (762)	0.1438	N/A	
PCP visits	144 (179)	158 (175)	141 (181)	116 (184)	<.0001	N/A	
Specialist visit	176 (472)	154 (537)	167 (345)	614 (1451)	<.0001	N/A	
Total medical	2080 (10 518)	1687 (5610)	1987 (11 254)	8247 (12 217)	<.0001	N/A	
Total pharmacy^c^	153 (602)	0 (0)	189 (614)	663 (1714)	<.0001	N/A	
Total surgical^c^	151 (3100)	0 (0)	0 (0)	5512 (17 969)	<.0001	N/A	
Total allowed costs^c^	2233 (10 544)	1687 (5610)	2176 (11 280)	8911 (20 119)	<.0001	N/A	
ET-related costs over total HCCs (%)	13% (2233/17 560)	13% (1687/13 255)	12% (2176/18 647)	30% (8911/30 130)	<.0001	N/A	

Abbreviations: ED, emergency department; ET, essential tremor; LOS, length of hospital stay; HCCs, healthcare costs; HCRU, healthcare resource utilization; N/A, not applicable; PCP, primary care provider. ^a^Unless otherwise noted, all data are reported as number (%) of patients. ^b^*P* value for comparison between overall patients with ET and those without ET. ^c^*P* value for comparison among treatment groups.

### Healthcare Costs

The average all-cause HCC was $17 560 per ET patient per year (PPPY), with ET-related HCC accounting for 13% ($2233 PPPY) of the all-cause HCC. Both all-cause and ET-related HCCs were higher among those in the invasive therapy group than in the pharmacotherapy and untreated groups (all-cause HCCs: $30 130, $18 647, and $13 255, respectively, *P* < .0001; ET-related HCCs: $8911, $2176, and $1687, respectively, *P* < .0001) (Table 4). All-cause and ET-related HCCs (PPPY) differed across demographic and clinical subgroups, including age group, gender, region, urban-rural residence, household income, payer type, number of comorbidities, presence of psychiatric disorders, and treatment type (all *P* < .0001) (**Supplemental Figures S4A and S4B; Supplemental Table S5**).

Multivariable analysis indicated age (*P* = .0032), region, living in an urban area (*P* < .05), higher household income (*P* < .05), commercial insurance (*P* < .0001), a higher number of comorbidities (*P* < .0001), presence of psychiatric disorders (*P* < .0001), and being in the invasive therapy group (*P* < .0001) were associated with increased all-cause HCCs. Similarly, older age, living in a suburban area, higher household income, a higher number of comorbidities, presence of psychiatric disorders, and being in the invasive therapy group were associated with increased ET-related HCCs (**Supplemental Figures S4C and S4D; Supplemental Table S5**).

### Comparison of Multimorbidity, Prevalence of Psychiatric Disorders, and HCCs Between Patients With and Without ET

**Figure 2.**
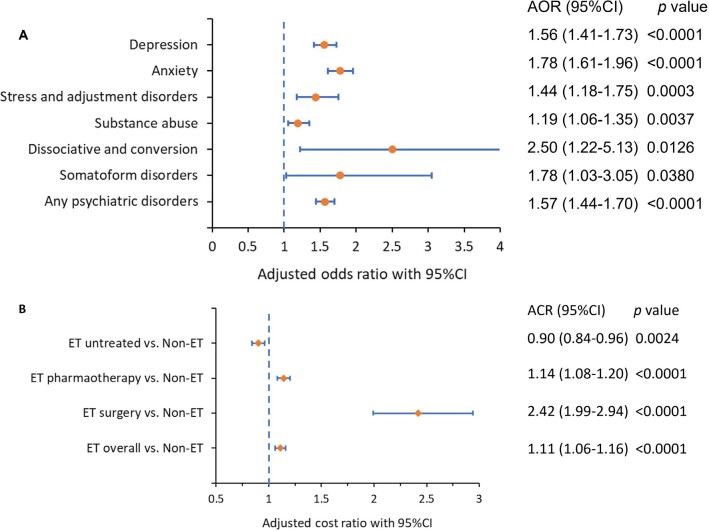
Forest Plot of Adjusted Odds Ratio of Prevalent Psychiatric Disorders (**A**) and Adjusted Cost Ratio (**B**) **(A)** Forest plot of adjusted odds ratio (OR) of prevalent psychiatric disorders during the 12-month follow-up period, ET vs non-ET. The vertical dashed blue line represents a OR of 1 as the reference line, which is associated with equal odds for both ET and non-ET groups. For each OR displayed, the solid orange dot depicts the OR, and the horizontal blue line represents the 95% CI. Lines that do not cross the dashed vertical blue line are statistically significant. **(B)** Forest plot of total all-cause costs accrued during the 12 month follow-up period, displayed as adjusted cost ratio (CR). The vertical dashed blue line represents a CR of 1 as the reference line, which is associated with equal costs for both ET and non-ET groups. For each CR displayed, the orange diamond symbol depicts the CR, and the horizontal blue line represents the 95% CI. Lines that do not cross the dashed vertical blue line are statistically significant. Abbreviations: CI, confidence interval; ET, essential tremor.

Over 99% of patients with ET (5244) had 1 matched patient without ET based on age, gender, payer type, first 3 digits of ZIP code, and index month. Patients with ET had more comorbidities (5.3 [SD = 3.2] vs 4.0 [SD = 3.3], Figure 1A); higher CCI (1.70 [SD = 2.29] vs 1.51 [SD = 2.22]); higher prevalence of psychiatric disorders (depression: 25.6% vs 15.3%, adjusted odds ratio (AOR) (95% CI): 1.56 (1.41-1.73); anxiety: 27.7% vs 15.5%, AOR: 1.78 (1.61-1.96); any psychiatric disorders (depression, anxiety, stress and adjustment disorders, dissociative and conversion disorders, somatoform disorders, substance use): 45.9 vs 31.1%, AOR: 1.57 (1.44-1.70) (**Table 3, Figure 2A**); and higher total all-cause HCC: $17 560 [$39 972] vs $13 237 [$27 098]. Adjusted cost ratio (95% CI) was 1.11 (1.06-1.16) (**Table 4, Figure 2B**); all *P* < .0001.

## DISCUSSION

This study is, to our knowledge, the first large-scale, population-based study evaluating the overall burden of comorbidities, psychiatric disorders, HCRU, and HCC among patients with ET in the United States and highlights the complexity of the ET population. The study demonstrated that hypertension (67.9%), pain disorders (61.8%), hyperlipidemia (55.4%), fatigue and sleep-related disorders (28.1%), and diabetes mellitus (27.3%) were among the most common comorbidities in patients with ET. The most prevalent psychiatric disorders were depression (27.7%), anxiety (25.6%), and substance use disorders (13.8%). The prevalence of comorbid pain disorders, fatigue, and sleep disturbances, as well as the increased prevalence of psychiatric disorders, highlight the mental health burden associated with ET. The increased prevalence of cardiovascular disease, diabetes, osteoarthritis, and obesity may be reflective of the older age of this population. Further studies are needed to better understand the relationship of these comorbidities with ET. Handforth and Parker^32^ proposed that chronic stress, including posttraumatic stress disorder, depression, and anxiety, may not only be responsible for the comorbidities associated with ET but in some cases may directly and indirectly induce ET. The rates of depression found among patients with ET in this study were lower than the rates reported in other studies. In this analysis, 25.6% of patients were diagnosed with depression, whereas 2 separate articles reported much higher rates.^33,34^ In one cross-sectional analysis in Spain, the self-reported depression rate was 43.8% in 235 patients with ET.^33^ Another cross-sectional study, including 245 Han Chinese patients with ET, assessed depression using the Hamilton Depression Rating Scale-24 Item and showed that 54.3% had at least mild depression.^34^ One possible explanation is that depression and other comorbid conditions such as fatigue, stress, and anxiety may not always cause the patient to seek care from a physician. Also, the studies from Spain and China included a small sample size, and the country, geographic region, race/ethnicity, and other factors could lead to the variation in the depression rate.

Our study demonstrated that 95.5% of patients with ET had at least 1 comorbidity, 79.7% had at least 3 comorbidities, and 56.9% had at least 5 comorbidities. Multiple comorbidities are associated with poor health outcomes: decreased quality of life,^32,35–37^ increased psychological distress,^38^ longer LOS, worse postoperative complications, and higher HCC and mortality.^24,39–41^ The interplay between multimorbidity and chronic disease is extremely important but receives inadequate attention in many chronic conditions.^24,41–43^ Data regarding the multiple comorbidities, their interactions, and the impacts on mental health and health economics among patients with ET are still scant.

Comorbidities increase the complexity of disease management and pose a significant clinical and public health challenge.^24,39–45^ Multiple comorbidities may lead to escalating use of multiple medications (polypharmacy), which may increase the risk of inappropriate medication use, accidental overdosing, poor medication or therapy adherence and persistence, adverse drug–drug interactions, and increased HCC.^24,43–45^ Pharmacotherapy is currently the mainstay for ET treatment.^13,14,46^ However, it has been reported that the first-line agents are ineffective in 30% to 70% of patients due to lack of response, adverse effects, and poor adherence and persistence.^11,13–15,46^ Our data show the wide spectrum of comorbidities among patients with ET, supporting the need for noninvasive, nonpharmacological treatments to minimize risk of adverse events. Novel treatment options can empower healthcare providers and ET care teams to provide safe and effective therapies that can be tailored to the specific healthcare needs of each patient. Because conventional clinical trials of devices, surgery, and medications in ET often exclude patients with some comorbidities,^20–23^ our data underscore the need for more inclusive pragmatic trials of all interventions in ET in the real-world setting.

This study indicates that patients with ET had higher HCC than the patients without ET: average all-cause HCC was $17 560 PPPY, ET-related HCC accounted for 13% of total HCC, and ET-related treatment significantly increased total HCC. Patient demographic and clinical factors associated with both increased all-cause HCC and ET-related HCC included number of comorbidities, treatment type, psychiatric disorders, age, geographic region, rural or urban residence. In addition, commercial health insurance was associated with increased all-cause HCC, and higher household income was associated with increased ET-related HCC. Invasive therapy, multiple comorbidities, and presence of psychiatric disorders remain the major drivers of HCC among patients with ET. Future studies are needed to focus on cost-effectiveness of novel therapies and new approaches in ET management in both commercially insured and Medicare Advantage populations.

### Limitations

There are several limitations in the study. First, this retrospective observational study using administrative healthcare claims data has inherent limitations because the data were collected for provider reimbursement and not for research purposes; therefore, certain comorbid conditions and outcomes may be underestimated. Second, since all comorbidities were identified based on ICD-10-CM codes alone, coding errors or misclassification are likely. However, this approach is commonly used in many other comorbidity studies.^24–29^ Third, there are no ICD-10-CM codes specific to the degree of ET severity or tremor location. Fourth, the study results may not be generalizable to the overall population, as those with other types of health insurance or uninsured may have different characteristics.

## CONCLUSIONS

While ET is traditionally regarded as benign, this study clearly highlights the significant impact of ET on patients, HCRU, and HCC. Multiple comorbidities and psychiatric disorders are highly prevalent among patients with ET and should be considered in the context of healthcare policy and medical decision-making to optimize ET care. Patients with ET are associated with substantial HCRU, posing a substantial economic burden to afflicted patients and the healthcare system. Invasive therapy, comorbidity burden, and presence of psychiatric disorders associated with ET remain major drivers for HCC. The data demonstrate increased mental health and HCC burden among patients with ET compared with matched patients without ET and underscore the need for innovation in care for this complex population.

### Disclosures

D.D, J.F., and H.C. are employees of CVS Health Clinical Trial Services LLC, which received funding from Cala Health, Inc to complete this research. A.S. was an employee of Cala Health, Inc at the time the study was conducted.

### Meeting Presentation

These data have previously been presented as posters at the International Society for Pharmacoeconomics and Outcomes Research, Virtual Annual International Meeting, May 17-20, 2021, and the 2022 American Academy of Neurology Annual Meeting, Seattle, April 2-7, 2022. The 3 abstracts were published in *Value Health.* 2021;24(suppl 1):S161; *Value Health.* 2021;24(suppl 1):S167; and *Neurology.* 2022;98(suppl):3207.

## Figures and Tables

**Figure:** Online Supplementary Material
